# PHF5A as a new OncoTarget and therapeutic prospects

**DOI:** 10.1016/j.heliyon.2023.e18010

**Published:** 2023-07-06

**Authors:** Xiaojiang Li, Dalong Liu, Yun Wang, Yu Chen, Chenyang Wang, Zhicheng Lin, Lin Tian

**Affiliations:** a Department of Orthopedics, Affiliated Hospital of Changchun University of Traditional Chinese Medicine, Changchun, 130000, China; b Department of Thoracic Surgery, Affiliated Hospital of Changchun University of Traditional Chinese Medicine, Changchun, 130000, China; c Department of Orthopedics, LiaoYuanCity TCM Hospital, LiaoYuan, 136200, China; d Department of Internal Medicine, Baishan Hospital of Traditional Chinese Medicine, Baishan, 134300, China; e Department of Lung Oncology, Affiliated Hospital of Changchun University of Traditional Chinese Medicine, Changchun, 130000, China

**Keywords:** PHF5A, SF3b, NSCLC, ESCs

## Abstract

PHF5A (PHD-finger domain protein 5A) is a highly conserved protein comprised of 110 amino acids that belong to PHD zinc finger proteins and is ubiquitously expressed in entire eukaryotic nuclei from yeast to man. PHF5A is an essential component of the SF3B splicing complex regulating protein-protein or protein-DNA interactions; particularly involved in pre-mRNA splicing. Besides its basic spliceosome-associated attributes encompassing the regulation of alternative splicing of specific genes, PHF5A also plays a pivotal role in cell cycle regulation and morphological development of cells along with their differentiation into particular tissues/organs, DNA damage repair, maintenance of pluripotent embryonic stem cells (CSCs) embryogenesis and regulation of chromatin-mediated transcription. Presently identification of spliceosome and non-spliceosome-associated attributes of PHF5A needs great attention based on its key involvement in the pathogenesis of cancer malignancies including the prognosis of lung adenocarcinoma, endometrial adenocarcinoma, breast, and colorectal cancer. PHF5A is an essential splicing factor or cofactor actively participating as an oncogenic protein in tumorigenesis via activation of downstream signaling pathway attributed to its regulation of dysregulated splicing or abnormal alternative splicing of targeted genes. Further, the participation of PHF5A in regulating the growth of cancer stem cells might not be ignored. The current review briefly overviews the structural and functional attributes of PHF5A along with its hitherto described role in the propagation of cancer malignancies and its future concern as a potential therapeutic target for cancer management/treatment.

## 1 Introduction

**Fig. 1:**
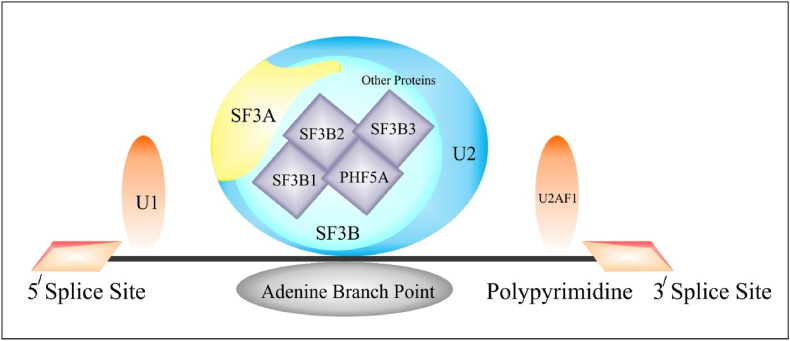
Schematic representation of PHF5A association with U2snRNP complex regulating its structural stability.

Cancer may broadly be defined as a term used to describe numerous disorders indicating the uncontrolled division of cells occurring due to the inability or abnormal functioning of basic regulatory mechanisms regulating the cell division in a controlled manner [1]. Cancer progression is a composite process occurring due to frequent genetic mutations with subsequent modifications of various molecular pathways that ultimately cause cell proliferation [2]. Several genes/proteins are known to be associated with cancer development occurring due to underlying gene mutations regulating the growth, apoptosis, and angiogenesis of cells [3]. Although several genes and numerous pathways are identified helping us to understand the network of a system, components of the network, and interaction among them, however only a few gene sets or oligonucleotides are considered essential for the persistence of the system [4,5]. Luckily, such essential genes are also reported to be effective drug targets in cancer treatment [[6], [7], [8]]. Essential genes primarily regulate fundamental cellular functions and from an evolutionary point of view are considered well-preserved than non-essential genes [9]. Complex nature of tumor microenvironment involving numerous proteins or genetic mutations introduced different metabolic pathways, their interconnected network and also explored different protein/gene targets specifically associated with progression and propagation of a particular cancer type. Essential genes perform particular functions while their altered functioning in carcinogenic cells also provoked their utilization as effective biomarkers and therapeutic targets (see Fig. 1).

Under nutrition deprived conditions tumor microenvironment relieves the cellular stresses and maintains the proliferation and progression of carcinogenic cells through exploitation of cellular stress response pathways leading to altered expression of transcriptional and translational regulatory pathways [10,11]. Cancer cells recruit mRNAs and ribosomes and effectively utilize unfolded protein response and mTOR signaling pathways to up regulate protein synthesis leading to improved tumor cells tolerability and pathogenesis. However, under nutrition starvation up regulation of numerous metabolic events particularly up regulated protein acetylation to regulate cell survival and proliferation meanwhile help the researchers to explore them as potential therapeutic targets [12,13]. Tumor microenvironment capability of sustaining cancer cells growth/proliferation under nutrition deprived conditions increased the interest researchers towards analysis of particular metabolic pathways involved in cancer propagation and selective targeting of such metabolic alterations or splicing manipulations for regulation and treatment of metastatic tumors.

Spliceosome machinery including both minor and major spliceosomes has been known to become associated with the regulation of pre-mRNA/RNA splicing and gene alternative splicing. Alternative/dysregulated splicing is a specific post transcriptional gene regulatory phenomenon responsible for proteome diversification through generation of multiple RNA transcripts from a single gene [14]. Tumorigenesis identified by the proliferation, metastasis and progression of oncogenic cells is greatly dependent on dysregulated/aberrant splicing of pre-mRNA splicing leading to the generation of aberrant proteins that accelerate carcinogenesis [[14], [15], [16]]. Spliceosome itself is a multi-megadalton protein-RNA complex mainly comprised of five small nuclear ribonuceoprotein (SnRNP) including U1, U2, U4/U6 and U5 and several non-SnRNP proteins with approximately 99.5% human introns being identified by the major spliceosome [[17], [18], [19]]. In pre m-RNA splicing U2 complex performs crucial role in regulation of alternative splicing through appropriate and precise recognition of the branch-point sequence [20]. SF3b complex is a basic functional subunit of U2 SnRNP and is comprised of SF3B1, SF3B2, SF3B3, SF3B4, SF3B5, SF3B6, and PHF5A/SF3B14b [21]. PHF5A is an essential splicing factors belonging to SF3B complex that presents pivotal involvement in alternative splicing regulation and cancer progression. Deep investigation of current biomarker is necessary to explore its role in cancer initiation and progression along with its future consideration as a suitable oncotarget in tumor therapy.

Cancer progression is considered to be associated with genetic mutation of cells and aberrant functioning of mutated genes including BRAF, EGFR, HER2, KRAS, PHF5A, MET, and TP53, etc. Modification of treatment strategies in a way of targeting these molecules/therapeutic targets plays an essential role in preventing the growth, progression, and metastasis of tumors [22]. Targeted therapy focusing on such specific molecules is considered to be highly effective as it ensures limited damage to the growth of normal cells and tissues. Moreover, every abnormal gene or molecule needs different therapy, therefore, enabling the clinicians about the possible treatment consequences and patient survival rate [22,23]. Utilization of novel techniques such as high throughput sequencing increased the knowledge of researchers towards the molecular level pathogenesis of cancer and also introduced new targets of molecular level associated with the prognosis of cancer [22]. Hence to achieve enhanced treatment outcomes and to improve patient survival the identification and targeted drug therapy targeting specific molecular sites is highly crucial [24]. Targeted drug delivery to particular molecular sites and improved patient survival to a certain extent in these cases have been observed during clinical practice [25]. Considering the associated factors current focus is to describe the targeted therapy for management of tumor malignancies using dysregulated alternative splicing of genetically altered signaling pathways. Current therapeutic system help the researcher to better understand and explore the underlying interconnected network responsible for the fabrication of carcinogenic cells along with identification of its network components and their intercommunication assisting the tumor cells growth, proliferation and invasion/migration to distant tissues/organs with its ultimate propagation to malignant form. Analysis of numerous oncogenic signaling pathways explored certain essential genes (proteins or oligonucleotides) that might be successfully employed as potential biomarkers for therapeutic management of malignant tumors.

PHF5A belongs to the PHD-finger genes superfamily encoding a protein comprised of 110 amino acids associated with the PHD zinc finger domain [26]. PHF5A initially described as a highly conserved and essential transcription regulator is a protein expressed in nuclei of all eukaryotic cells [27]. Accordingly, PHF5A protein is described as a vital constituent of the splicing factor 3b (SF3b) complex that consequently is a constituent of RNA spliceosomal machinery [28]. PHF5A not only directly regulates protein-protein interactions via RNA splicing pathway but also regulates numerous non-spliceosome associated functions [29] such as involvement in cell cycle regulation [30], cellular growth and differentiation [31], DNA repair [32], morphological growth and development of cells, tissues, and organs [33], chromatin remodeling [29], and embryonic stem cells pluripotency subsistence [34]. Abnormal functioning of PHF5A (PHD-finger domain protein 5A) is associated with proliferation, invasion, and metastasis of tumor cells; biologically a malignant characteristic of cells [35,36].

Hence, PHF5A is an important subunit of U2 snRNPs playing pivotal role in regulation of pre-mRNA splicing/alternative gene splicing with resultant improved tumor progression, propagation and metastasis based on specific branch point sequence recognition. Hyperacetylation of PHF5A is considered to be associated with improved tumor pathogenesis that ultimately increased our attention towards therapeutic prospective keeping in view PHF5A as novel oncotarget for tumor treatment. PHF5A consideration seems highly appealing as a potential drug target for site specific irreversible eradication of carcinogenic cells attributed to its pivotal participation in regulation of both spliceosome and non-spliceosome associated attributes of CSCs. PHF5A targeting might be helpful in identification of diverse splicing pathways and some interconnected extremely sheltered splicing factors responsible for tumor progression through undefined molecular mechanisms. All such information may help the researcher in the designing of therapeutic agents exhibiting site specific pharmacodynamics effects along and synthesis of innovative therapeutic substitutes from existing chemotherapeutic moieties.

## 2 Structural attributes of PHF5A

PHF5A is a structurally intact gene encoding an immensely preserved diminutive protein comprised of 110 amino acids exhibiting a molecular weight of 12,405 Da; acquiring a specific PHD-finger domain [26]. Hitherto in 1993, the PHD-finger domain was expounded by Schindler et al. within HAT3.1 homeobox protein with its recognized sequence comprised of eight uniformly spaced amino acids i.e. [Cys_4_–His–Cys_3_] [26,37]. PHD-finger proteins are mainly intricate with (a) chromatin-assisted transcriptional regulation via chromatin transforming complexes [38,39] and (b) transcriptional activation and repression via transcription factors and cofactors. PHF5A protein expression (although initially identified in the E territory of mouse chromosome 15) is noticed in nuclei of almost all eukaryotes comprising of yeast, plants, and mammals including human beings with the operational territory being associated with protein-protein or protein-DNA interaction [40,41]. The uniformity/similarity among coding sequences of rat/mouse PHF5A and PHF5A of human beings are identified to be 91% and 94% respectively however, absolute uniformity/similarity is detected in amino acid sequences of the proteins associated with such species [26]. Hence, for structural point of view PHF5A is an extremely conserved protein that corresponds to PHD-finger domain and is expressed in nuclei of almost all eukaryotes where its expression is associated with chromatic remodeling and transcriptional factors/co-factors activation and suppression.

## 3 Biological attributes of PHF5A

PHF5A is recognized to regulate a wide range of biological attributes primarily classified into spliceosome-associated and non-spliceosome-associated attributes [26].

### 3.1 PHF5A role in the regulation of spliceosome-associated attributes

Splicing, probably a principal way of transcriptional modification is a complex phenomenon of transforming precursor mRNA or pre-mRNA into mature mRNA owing to immensely specific and stepwise interactions [42]. Alternative splicing involves the transformation of pre-mRNA into m-RNA isoforms in such a way that maturely spliced m-RNA variants exhibit quite discrete structural and functional attributes thus providing transcriptional diversity to proteome through various splicing tactics [43]. Alternative splicing plays an enormously pivotal role in the diversification of intracellular proteins along with the regulation of cellular homeostasis and organization of cellular growth and differentiation and tissue/organs formation [44,45]. Alternative splicing occurs in approximately 95% of human genes inducing variable degree alterations in a cellular nucleoplasm [44].

In contrast, dysregulated or abnormal splicing associated with the malfunctioning of maturely spliced m-RNA variants results from the interference of various exogenous or endogenous factors with splicing, spliceosome complex, or splicing factors [46]. Abnormal or dysregulated splicing is considered to be associated with the formation of malignant protein isoforms that ultimately provoke cancer formation in the human individual [47,48]. Activation of abnormal splicing may activate cancer-promoting functions through input in several biological processes leading to the initiation and proliferation of carcinogenic cells, tumor development, drug resistance, etc. A group of splicing modulators particularly targeting SF3b complex (or its PHF5A component) are illustrated to display cytotoxic activity against cancer cells and might be considered potential therapeutic targets for the management of cancer [29]. Numerous hitherto studies signified the role of PHF5A in the initiation and progression of the lung, breast, and colorectal cancers [35,36,[49], [50], [51]]. Though alternative splicing has been identified as a suitable mean of attaining structural/functional diversity in proteins basically required for maintaining cellular growth/differentiation and homeostasis. However aberrant/dysregulated splicing occurring from the snooping of different spliceosomal factors/co-factors may provoke cancer progression and propagation attributed to the formation of abnormal protein isoform responsible for over expression of different splicing modulators such as PHF5A. Luckily overexpression identification of such biomarkers open another mode of site specific therapy based on utilization of theses biomarkers as a potential drug targets in cancer therapy.

Alternative splicing is assembled by a range of cellular spliceosome components reported as nuclear small ribonucleoproteins among which U2-snRNP or U2 small nuclear ribonucleoprotein is a dynamic splicing factor acting as a cellular tool liable for separating introns from pre-mRNAs [52]. PHF5A is a fundamental constituent constituting U2-snRNP complexes that regulates U2-snRNP and SF3b recruitment and structural stability [29]; SF3B is a key component (or sub-complex) of U2-snRNP comprised mainly of yeast proteins [52,53]. SF3b, SF3A, and snRNA 3ʹ binding proteins particularly PHF5A are subcomplexes of splicing factor 3 (SF3) complex primarily involved in U2-snRNP composition, assembled via the interaction of U2-snRNAs with companion proteins [52,54,55]. In RNA splicing; PHF5A ensures sequence accuracy by pinpointing the branch point sequences at the 3′ end splice site [20]. A study performed by Rzymski et al. (2008) described PHF5A functioning as a bridging factor that functions to assist the association between proteins of splicing factors and ATP-dependent helicases. PHF5A facilitates the association of U2 snRNP with DDX1 (ATP-dependent RNA helicase) and EP400 (ATP-dependent chromatin remodeling helicase) initiating binding via its N-terminus [56]. Furthermore, mouse PHF5A analysis indicated its robust expression in spermatocyte-specific cell lines with variable level expression at various stages of mouse spermatogenesis [57]. During mouse spermatogenesis, the co-occurrence and coordinated expression of PHF5A with U2AF1 splicing factor in primary spermatocytes provoked it as a pivotal factor for meiotic differentiation of male germ cells [56,57]. PHF5A is identified as a functional constituent mainly responsible for constitution, recruitment, composition of U2-snRNP along with structural stability maintenance of U2-snRNP and SF3b. Further PHF5A role in regulation of sequences accuracy through precise recognition of branch point at the 3′ end splice site in RNA splicing has also been described.

### 3.2 PHF5A participation in the regulation of non-spliceosome-associated attributes

Non-spliceosomal functions of PHF5A are associated with the SF3b component of U2snRNP and include, PAF1C transcriptional complex recruitment and stability, meticulous delineation of adult myoblasts, pluripotency regulation, pluripotent embryonic stem cells differentiation and execution of RNA polymerase II elongation dynamics at pluripotent localities [34]. Further PHF5A also contributes to DNA repair and direct histone modifications [58]. Numerous cell-specific transcriptional factors such as PHF5A present a key role in ESCs regulatory networks assisting ESCs to self-renew and differentiate into all tissues during embryonic development; thus orchestrating the stability of gene expression [34]. Appropriate preservation of different pluripotent regulatory factors such as Oct4, Sox2, and Nanog stimulate Embryonic Stem Cells' capability of indefinite proliferation, self-renewal, and multi-directional differentiation causing differentiation of cells in virtually all cell types [59,60]. Polymerase associate factor 1 complex (PAF1C) is an essential component that directs gene expression or transcription via regulation of (a) deposition of histone modifications, (b) RNA polymerase II functions, and (c) cellular reprogramming or cell-specific proliferation and differentiation of embryonic stem cells [61,62]. Accordingly, PHF5A assisted ESCs pluripotency and self-renewal phenomenon indicated its hindered role in PAF1C the structural stability maintenance, regulation of RNA polymerase II elongation at pluripotent localities, and chromatin binding [21]. Briefly, PHF5A contribution to the regulation of non-spliceosomal functions includes DNA damage repair, ESCs pluripotency/cellular growth or differentiation maintenance, RNA polymerase II chain elongation at pluripotent sites and PAF1C regulation.

In contrast, PHF5A depletion or knockdown negatively affects pluripotent gene elongations triggering ESCs differentiation with eventual inhibition of ESCs pluripotency and cellular reprogramming. Further, obtained findings verified reduced interaction or interaction loss among PHF5A and PAF1C subunits determined via mass spectrometry and co-immuno precipitation techniques [21]. Similarly, PAF1C stability upon PHF5A silencing was also analyzed using density sedimentation analysis both in the presence and absence of PHF5A. Results indicated PAF1C distribution into low molecular weight fragments upon PHF5A depletion suggesting its destabilization with the consequent inability of binding to its target genes; thus certifying PHF5A as a strong binding partner of PAF1C [34]. Distorted transcription and aberrant elongation of RNA polymerase II owing to PAF1C loss due to PHF5A knockdown has been reported. PHF5A depletion with respective peculiar loss of PAF1C was perceived to be associated with deposition of H2B ubiquitylation indicating a pronounced increase in H2BK120 deposition along with reduced H3K79methylation; two vital histone modification markers regulating transcriptional pluripotency [63].

Corresponding analysis of PHF5A expression in pluripotent ESCs and differentiated tissues at both gene and protein levels revealed its pronounced expression in ESCs at both levels [21,64]. However, PHF5A silencing displayed up-regulated expression of PAf1C on gene bodies in early embryonic development along with downregulation of self-renewal markers in differentiated tissues or adult ESCs. Similarly, the halt and expansion of proximal promoters of pluripotent ESCs upon PHF5A depletion have also been reported as responsible suggesting its assistance in transcriptional regulation of ESCs pluripotency, self-renewal and cellular differentiation [34,64]. Reported studies also narrated the role of PHF5A in chromatin modeling describing it as a protein of chromatin-modulating complexes [26,34].

PHF5A has also been described as a splicing factor modulating DNA damage repair via the retention of chromatin integrity. DNA breaking repair associated with non-homologous end assembly and maintenance of chromatin integrity is dependent on the formation and deposition of histone H2A variants including H2AX and H2AZ. DNA damage requires a preliminary collection of proteins at the DNA damage site which is regulated through the deposition of the H2AX variant. PHF5A is reported to be indirectly involved in DNA damage repair response through regulation or stabilization of histone 400 chaperone complexes; perceived in mouse B cells [32].

**Fig. 2:**
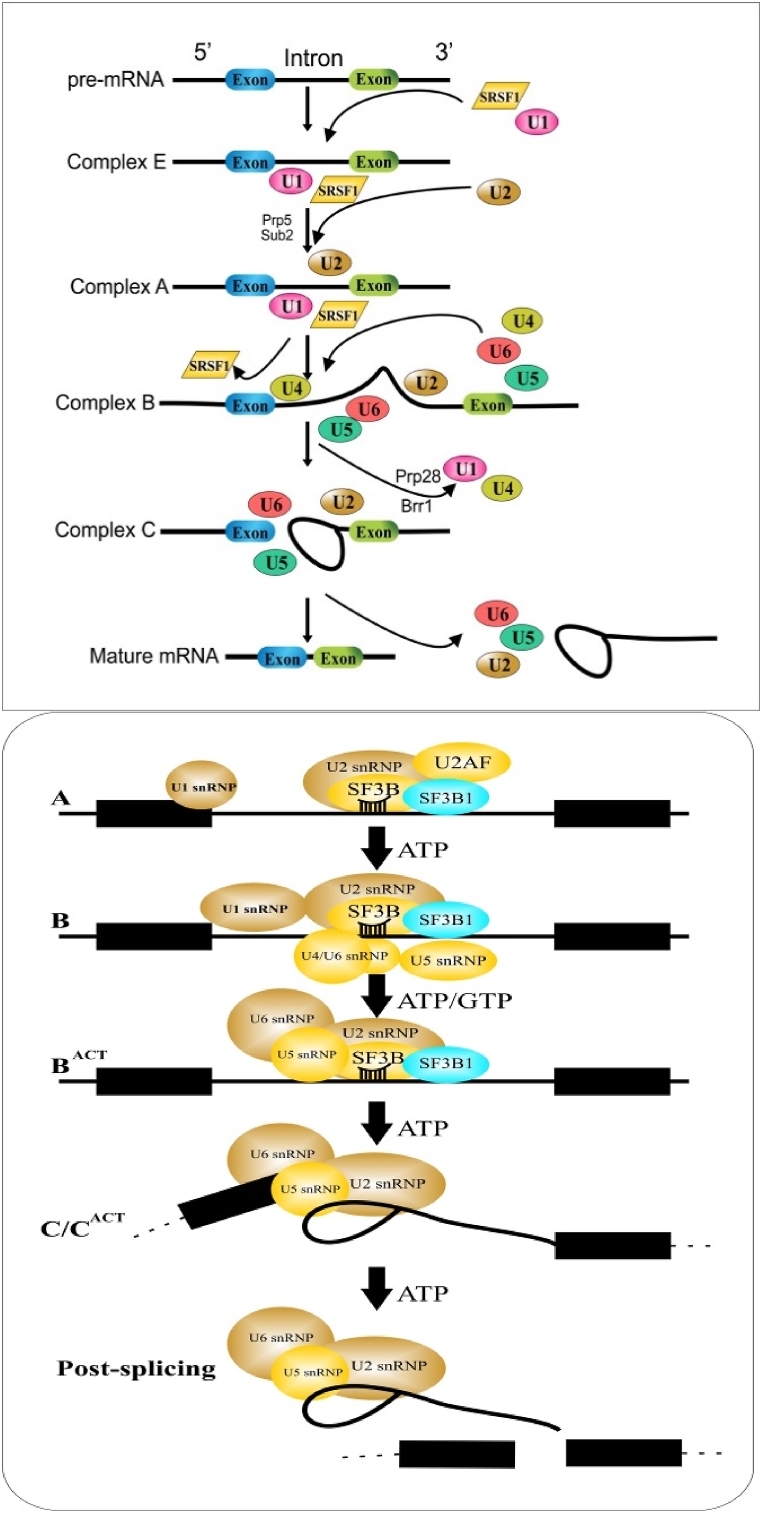
Schematic demonstration of pre m-RNA splicing to mature m-RNA via major spliceosome factors and representation of SF3b1-complexes during spliceosome assembly.

PHF5A seems to indicate tissue and time-specific expression during the embryonic development of *Caenorhabditis elegans*. PHF5A expression was initiated at the morphogenetic phase of embryogenesis with its broad expression in comma-staged embryos and later stages of embryonic development [65]. Here PHF5A expression was linked to organogenesis and muscular development attributed to muscle-specific expression and regulation of intracellular adhesion proteins. Larvae death resulting from the diminution of PHF5A protein probably corresponds to specific dysfunctioning of the pharyngeal, body wall, and anal muscles [26,33]. Limited PHF5A expression in later developmental stages was also reported in adult animals indicating its remarkable expression in the pharynx only. PHF5A gene silencing or knockdown resulted in absolute embryonic lethality signifying its pivotal role in morphogenetic and organogenesis phases of embryonic development [33]. In yeast, the lethal effects resulting from PHF5A protein depletion have also been reported however, the underlying mechanism of its functioning in embryogenesis and histomorphological development needs further explication [26]. In short PHF5A silencing or knockdown provoked negative effects on non-spliceosome attributes such as suppression of PHF5A and PAF1C subunits interaction, abnormal RNA polymerase II chain elongation, suppression of ESCs pluripotency, self-renewal and cellular differentiation, inhibitory effect on regulation or stabilization of histone bodies (histone 400 chaperone complexes) required for DNA damage recovery together with lethal effects on embryonic development such as inhibition of embryogenesis (see Fig. 2).

## 4 PHF5A participation in cancer progression

**Fig. 3:**
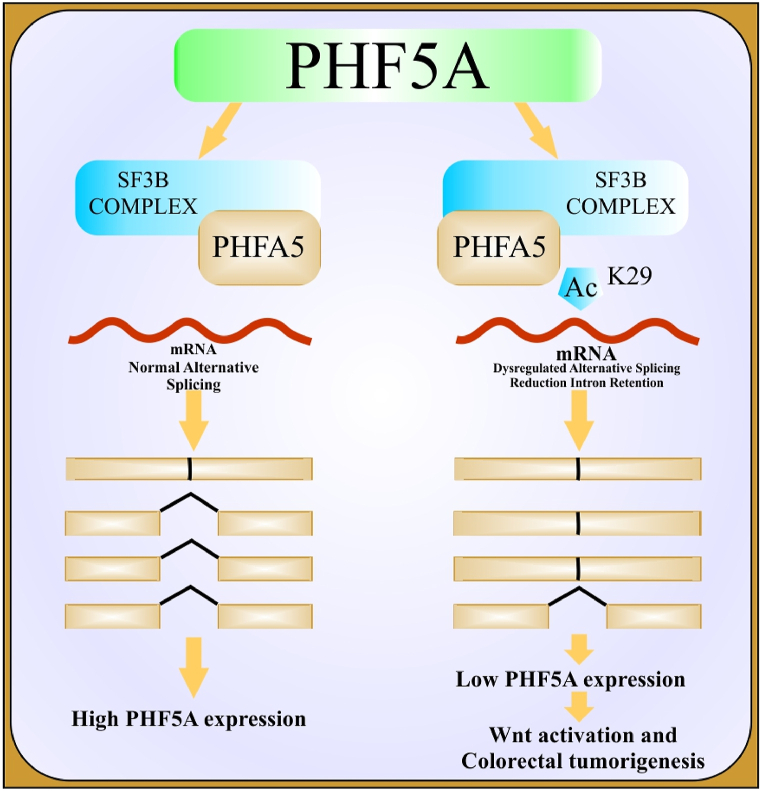
Figure indicating the role of PHF5A in the progression of colorectal tumor via activation of Wnt signaling pathway.

**Fig. 4:**
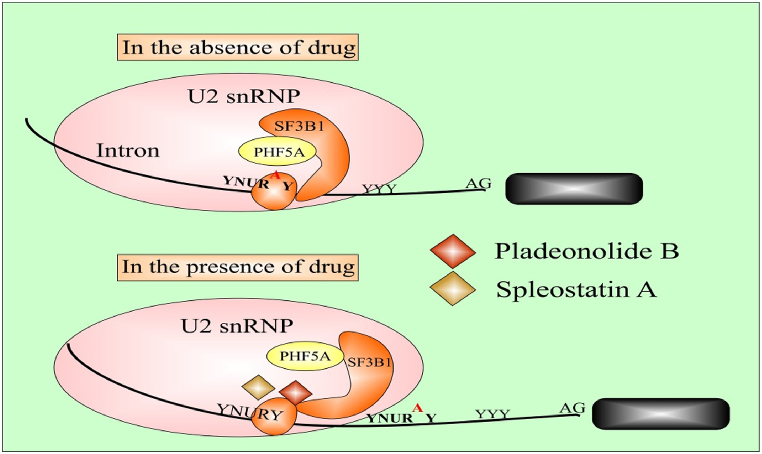
Diagrammatic representation of splicing changes induce through splicing modulators particularly antitumor drugs that bind with and interfere with SF3B1 and PHF5A subunits of the U2 snRNP.

Alternative splicing and its regulation via splicing factors play a pivotal role in the metastasis of cancer. Regulation of abnormal genes most frequently results in the incidence and proliferation of cancers [46]. Currently, PHF5A participation in the incidence and proliferation of numerous cancers like lung cancer, breast cancer, colorectal tumors, endometrial tumors, squamous cell carcinoma, oral cell carcinoma, and hepatocellular carcinoma has been elucidated [66]. Accordingly, PHF5A regulates the activation of proto-oncogenes/proteins through dysregulated or abnormal alternative splicing of different transcription factors and cofactors [35,36,49,[67], [68], [69]]. Further, PHF5A working in the regulation of ESCs growth and self-renewal has also been described hitherto [31,64]. Table 1 and Fig. 3 briefly summarized the biological and regulatory attributes of PHF5A in different cancers (see Fig. 4).

**Table 1:** Biological and Functional attributes of PHF5A in progression of Cancer.

Tumor type	Function	Targeted gene/protein	Regulatory Mechanism/pathway	Outcomes	References
**Breast cancer**	Splicing factor	FASTK	Regulation of the FASTK-AS axis as an epigenetic inhibitor of apoptosis	Enhanced cell proliferation, migration and tumorigenesis, inhibition of cell apoptosis	Zheng, Y.-Z. et al. (2018) [33].
**Non-small cell lung cancer and its cancer stem cells**	Oncogenic factor; Splicing factor	IGFBP3, DDIT3, CHD4 and HDAC8, PIK3CB, SKP2, etc.	PHF5A-TOMM22-oxidative phosphorylation regulatory network; CHD4-PHF5A interaction activates RhoA/ROCK pathway	Enhanced cell proliferation, migration, invasion and cancer pathogenesis, inhibit cell apoptosis, promoted xenograft tumor growth; tumor progression and poor patients survival	Mao et al. (2019) [47] Nuo et al. (2020) [70] Yang, Y. et al. (2022) [71].
**Lung Adenocarcinoma cells**	Oncogenic factor; Splicing factor	IGF-1 pathway effector genes (IGFBP3, PIK3CB, and AKT2)	Multiple signaling pathways	Enhanced cancer cells proliferation, migration, invasion and vice versa results upon PHF5A suppression	Yang Y et al. (2018) [69].
**Colorectal cancer**	Splicing factor	KDM3A、TEAD2	Enhance the interaction between U2-nRNP subunits and affect the overall splicing pattern of pre-mRNA; high PHF5A-K29 acetylation-induced AS up-regulates KDM3A and activates Wnt pathway, thereby regulating cellular stress response; promotes TEAD2 exon 2 inclusion body Splicing to activate Yes-associated proteins	Promoted cell proliferation and metastasis in-vivo and in-vitro; promoted xenograft tumor growth; closely related to poor clinical stage and low 3-year survival rate of patients	Wang et al. (2019) [34]. Chang et al. (2021) [48].
**Hepatocellular carcinoma**	Oncogenic factor	NF-kB	Enhance NF–B pathway activity, upregulated MMP9 and Slug	Promoted the migration, invasion and progression of hepatocellular carcinoma	Yang, Q. et al. (2019) [65].
**Stomach cancer**	Oncogenic factor	NF-kB	Enhance NF-kB pathway activity, up-regulate phosphorylated 1Bx and cyclin DI	Promoted cell proliferation and migration along with poor patients survival	Zhang, Z. et al. (2023) [72] Cao et al. (2020) [73].
**Oral squamous cell carcinoma stem cells**	Splicing factor			Reduced disease-free survival of patients	Mohanta, S., S.S. Khora, and A. Suresh. (2019) [67].
**Pancreatic cancer stem cells**	Splicing factor		Form PAFI-PHF5A-DDX3 subcomplex, locate in the Nanog promoter region, increase the expression of transcription factors and stemness markers of tumor stem cells in order to maintain stemness	Assists the formation of tumor spheres, tumor formation and their metastases	Karmakar, S. et al. (2020) [62].
**Endometrial cancer**	Splicing Factors/Co- Factors	Connexin43 gene with subsequent stimulation of AF-1 (transcriptional activating factor-1), connexin α1gene expression	In the presence of estrogen, the expression of Gjal is promoted	Associated with endometrial adenocarcinoma	Oltra et la. (2003) [25]. Falck, E. and K. Klinga-Levan. (2013) [66].
**GBM stem cells**	Splicing factor		Enhanced recognition of C-rich 3′ splice site exons	Promote the proliferation of GBM stem cells and the occurrence and development of xenograft tumors	Hubert, C.G. et al. (2013) [29]. Sato, M. et al. (2014) [74].

### 4.1 PHF5A accelerates the progression of cancers via regulation of alternative splicing of target genes

PHF5A was initially described as an essential splicing factor actively participating as an oncoprotein in the progression of breast cancer and might be pondered as a potential therapeutic drug target [72]. PHF5A was identified as a potential epigenetic inhibitor of apoptosis that was over-expressed in breast cancer with simultaneous association to deprived survival [35]. *In-vitro* PHF5A silencing depicted overexpression of FASTK protein that assisted FAS-mediated apoptosis with ultimate suppression of cancer cell proliferation, invasion, and progression *in-vivo*. High expression of FASTK was verified through *in-vitro* analysis of breast cancer cell samples primarily of triple-negative subtypes [35].

The oncogenetic role of PHF5A role in lung cancer progression has recently been narrated by Mao et al. (2019). TCGA and tissue microanalysis indicated the significant over-expression of PHF5A in cancer cell lines upon investigation of the clinical and biological performance of PHF5A in NSCLC (non-small cell lung cancer) [49]. High expression PHF5A was associated with upregulated proliferation, invasion, and metastasis of cancer (LAUD) cells and prohibition of cisplatin provoked apoptosis with subsequent poor survival of lung adenocarcinoma patients [49].

Similar findings reporting PHF5A as oncoprotein and potential prognostic factor and potential therapeutic target has also been reported by Yang Y et al. (2018). Here the upregulated expression of PHF5A resulting from the aberrant regulation of multiple signaling pathways was correlated with enhanced proliferation and progression of lung adenocarcinoma cells in comparison to adjacent non tumor tissues [73]. In a former study the fundamental role of PHF5A in alternative splicing of various down-regulated genes particularly apoptosis-related (API5 & BCL2L13) and cell cycle-related splicing factors (including SKP2, CHEK2, ATR) has been identified. Moreover prohibition of LAUD cells proliferation, invasion, and migration associated with PHF5A Knockdown and pladienolide (small molecular prohibitor of PHF5A inhibitor) incorporation has also been investigated that provoked PHF5A biomarker as a potential therapeutic target for cancer treatment malignancies [49].

Nutrient starvation induces multiple cellular stresses correlated to the hyperacetylation of different proteins. Multiple cellular stresses are also known to provoke acetylation of p-300-dependent PHF5A K-29 (Lysine 29 residue) [36]. Upregulation of PHF5A K-29 causes over-expression of KDM3A via dysregulation of its pre-mRNA alternative splicing leading to colorectal tumorigenesis through activation of the Wnt signaling path. The underlying mechanism suggested that multiple cellular stresses signify enhanced interaction of PHF5A-K29 with U2-snRNP-associated proteins altering the overall splicing pathway of pre-mRNA and associated modified genomic expression [36].

Likewise, Chang et al. (2021) also narrated the positive involvement of PHF5A in the progression of colorectal tumors via epigenetic modification of protein acetylation [50]. Comparative analysis of PHF5A expression in colorectal cancer cells and normal paracancerous cells indicated its over-expression in tumor tissues. Up-regulation of PHF5A was associated with enhanced migration and invasion of tumor cells together with poor survival and disease-free clinical outcomes of patients with colorectal cancer. Stimulation of the YAP signaling pathway leading to aberrant regulation of alternative splicing of TEAD2 gene exon 2 to its full-length isoform (TEAD2-L) was described as the suggested tumorigenesis mechanism with ultimate enhanced proliferation, metastasis, and progression of colorectal tumor. Moreover, the over-expression and partial involvement of PHF5A- H3K27 in the progression of colorectal cancer have also been demonstrated [50].

PHF5A participation as a promising oncoprotein inducing epigenetic modification of protein acetylation or hyperacetylation in tumor pathogenesis has been well described hitherto. Thus, PHF5A consideration as a potential therapeutic target in cancer treatment based on its pivotal oncogenic regulation of cancer cells proliferation, invasion, migration. Aberrant splicing and epigenetic modifications of numerous signaling pathways and target genes provoke PHF5A as an important therapeutic biomarker. Further, PHF5A knockdown or silencing coupled with concomitant administration of conventional chemotherapeutic agent might also be a suitable site specific/targeted therapeutic option for cancer therapy.

### 4.2 PHF5A participation as oncogene or proto-oncogene in cancer progression

PHF5A is reported to contribute well as a proto-oncogene in the progression of different malignancies including breast cancer, lung cancer, gastric cancer, hepatocellular carcinoma, endometrial adenocarcinoma, etc. [67,68,70]. Overexpression of PHF5A along with T lymphocyte infiltration and reduced survival of patients displaying gastric cancer was noticed. PHF5A knockdown indicated the prohibition of cancer cells via the AKT/mTOR signaling pathway with eventually enhanced apoptosis and reduced proliferation/migration of cancer cells [70]. Similar findings were also reported in a research study conducted by Cao et al. (2020). High expression of PHF5A in gastric carcinoma cells (MGC 803 cells) specified promoted tumor pathogenesis attributed to stimulation of NF- κB signaling pathway while PHF5A knockdown presented negative consequences i.e. prohibition of gastric carcinoma cells proliferation, migration, and prognosis [74]. Overexpression of PHF5A in hepatocellular carcinoma cells has also been reported with its depletion being associated with reduced invasion, migration, and propagation of hepatocellular carcinoma. Progression of hepatocellular carcinoma was correlated to PHF5A-mediated activation of the NF-***κ***B signaling pathway since PHF5A depletion indicated downregulation of the current/classical signaling pathway along with the prohibitory impact on tumor prognosis [67].

Similarly, Yang et al. (2018) also explored the pivotal attributes of PHF5A as an oncoprotein associated with the poor survival of lung adenocarcinoma patients. The current study explored the oncogenic role of PHF5A in the pathogenesis of lung adenocarcinoma via the regulation of numerous signaling pathways including IGF-1. PHF5A upregulation was associated with an increase in tumor size, enhanced lymph node metastasis, and pathogenesis of tumor to advanced-stage malignancy. PHF5A silencing or depletion provoked vice versa outcomes indicated by promoted apoptosis and cell cycle arrest coupled with reduced invasion, migration, or proliferation of lung adenocarcinoma cells. Again the promising therapeutic potential of PHF5A in the management of cancer malignancies was acknowledged [51]. The pivotal role of the CHD4 protein in DNA damage repair and regulation of the cell cycle is well established. However, it’s functioning in the proliferation and prognosis of cancer was not thoroughly illuminated.

Nuo et al. (2020) noticed the over-expression of CHD4 (chromodomain-helicase DNA binding protein 4) in NSCLC (non-small cell lung cancer) concerning its adjacent normal tissues. Progressive tumor prognosis indicated by enhanced growth, migration, and proliferation of NSCLC cells was correlated to the over-expression of CHD4 along with deprived survival of patients. Molecular analysis for fundamental mechanism identification suggested its promising interaction with PHF5A along with successive stimulation of the RhoA/ROCK signaling pathway. Further, CHD4 silencing or knockdown provoked expected outcomes i.e. suppression of tumor proliferation and migration via cell cycle cessation at the G1/S phase [75]. PHF5A participates well in progression of different types of cancers including breast, lung, hepatocellular, lung adenocarcinoma and gastric tumors occurs through regulation/stimulation of numerous signaling pathways such as AKT/mTOR, NF- κB, IGF-1 and YAP signaling pathways. Interconnected communicated network of different signaling pathways associated with up regulated expression of different splicing factors or biomarkers ultimately help the tumor microenvironment to sustain carcinogenic cells growth and proliferation under starved conditions via energy/ATPs generation from multiple pathways.

### 4.3 PHF5A participation in cancer prognosis or pathogenesis as transcriptional factor/co-factor

The potential functioning of PH5FA as a nuclear transcription factor/co-factor in the prognosis of cancer was described by Oltra and his colleagues (2003). PHF5A upregulation was perceived to augment estrogen response to the cx43 gene (connexin43) in a concentration-dependent manner. The process was initiated via binding of PHF5A to the promoter region (−74 to −34 nucleotides) of the cx43 gene also called connexin43 with subsequent stimulation of AF-1 (transcriptional activating factor-1) function of estrogen receptors with a resultant improved response of estrogen to cx43 gene. Upregulation of PHF5A with subsequent connexin α1 gene overexpression and stimulation of intracellular transmission has been detected in cervical cancers [27]. Similarly, to determine the expression level of connexin α1gene and PHF5A in human and rat adenocarcinoma cells Falck et al. (2013), performed a comparative analysis. Here, connexin α1gene and PHF5A expression in both rat and human endometrial adenocarcinoma cells were determined and compared to their expression in benign cancer tissues. Results indicated up-regulated expression of connexin α1gene and PHF5A in human endometrial cells with its down-regulated expression in rat endometrial cells in comparison to benign cancer tissues [68].

Thus, expression of PHF5A as transcriptional factors inducing enhanced stimulation of AF-1 and Connexin α1gene provoke its diverse oncogenic functioning in endometrial cancer pathogenesis however, the pathophysiological operation of PHF5A along with its underlying mechanism needs advanced investigation at molecular level.

## 5 PHF5A contribution to the regulation of cancer stem cells and its underlying mechanism

Initial tumor onset, growth, and prognosis occur due to the presence of particular self-renew type cells also called cancer stem cells (CSCs) exhibiting specific attributes enabling them to propagate via self-renewal, invasion or migration, indefinite proliferation, and potential of differentiate to produce site/function specific mature cells [76]. Cancer Stem cells also contribute to the repeated occurrence, metastasis, and potential resistance after the tumor management hence, removal of these cells is a prerequisite demand to resolve the clinical crises [76,77].

Tumor malignancy may simply be described as the formation of an aberrant organ by a subset of cells (cancer stem cells) that displays the capability of unlimited proliferation through accumulated mutations developed by cellular division over a prolonged time [78]. Homologous attributes of normal and tumorigenic cells may explain the mechanism of tumor development that includes the proliferative capability of both types of cells to divide and form new tissues [79,71]. Correspondingly, both types of tissues are comprised of a diverse combination of cells with variable proliferative prospective and phenotypic characteristics [79]. Similarly, both types of cells exhibit the capability of differentiation to form mature cells/tissue however, In contrast to normal cells cancer cells present some sort of heterogeneity arising from continued mutagenesis due to aberrant differentiation of tumor cells. Such abnormal expression of normal differentiation markers ultimately led to tumor pathogenesis [76,79,80].

PHF5A not only participates in the regulation of tumor pathogenesis nevertheless but its contribution in regulating the growth and self-renewal of stem cells is also stated [31,64]. PHF5A also acts as a key regulator required for the enhancement of glioblastoma multiforme stem cells; identified using multigenome wide RNAi screens derived from patient glioblastoma multiform stem cells and compared to neutral stem cells and fibroblasts. PHF5A knockdown in glioblastoma stem cells indicated prohibition of PHF5A-assisted splicing genes and associated U2 SnRP activity with consequent cell cycle arrest and viability loss. Correspondingly, PHF5A silencing prevented the growth of conventional patient-originated glioblastoma stem cell xenografts along with *the in-vivo* prohibition of glioblastoma stem cells tumor growth [31]. In pancreatic cancer stem cells, the interaction of PHF5A with PAF1C and DDX3 led to the overexpression of Nanog and other associated genes responsible for stemness genes regulation. Hence, the potential targeting of the PAF1-PHF5A-DDX3 complex might be beneficial in preventing or reducing the prognosis of pancreatic cancer [64].

PHF5A has been described to act as a promising cancer stem cell (CSCs) biomarker/prognosticator predicting the prognosis of cancer stem cells in a patient with oral carcinomas i.e. squamous cell carcinoma. Comparative analysis of micro-array-based meta-analysis and TCGA/CSC database provoked the pivotal contribution of PHF5A to deprived disease-free persistence of patients with oral cancers [69]. Overexpression of PHF5A was associated with expanded proliferative potential and increased invasive/migratory aptitude of NSCLC permitted to stimulation of stemness phenotype. In contrast, PHF5A knockdown or depletion provoked reverse outputs with inhibition of NSCLC proliferation, migration, or invasion corresponding to the prohibition of such phenotype [81]. Bonnal et al. (2020) recently reported frequently occurring splicing mutations that ultimately results in cancer malignancies along with its prevalence rate. Their findings are summarized in Table 2 [82].

**Table 2:** Splicing-factor mutation frequently encountered in cancer malignancies and its associated prognosis.

Splicing factor	Cancer type	Prevalence (%)	Prognosis	References
**SF3B1**	CLL	5–31	Shorter OS PFS and TTT (when clonal: variant allele frequency >12%); or no effect on OS, PFS or ORR	[83,84].
	MDS without RS	7	No effect on PFS, AML transformation or OS	[85].
	Primary myelofibrosis	6.5	No effect on OS	[86].
	MDS with RS	16–77	Longer LFS and OS	[87].
	MDS	7–81	Lower cumulative incidence of disease progression, longer LFS(u), EFS and OS (u); no effect on OS	[88,89].
	Mucosal melanoma	22	Shorter PFS and OS	[90].
	Primary orbital melanoma	36	Tendency for longer OS (in a small cohort of patients)	[91].
	De novo AML	2.4	Shorter OS and DFS, and lower complete remission rates	[92].
	Breast cancer	5–10	Shorter OS (luminal B and progesterone receptor-negative disease)	[93].
	Uveal melanoma	15–22	Longer EFS and cancer-specific survival; late onset metastases, intermediate risk of metastases, but worse DFS in disomy 3 group; tendency for longer OS (in a small cohort of patients)	[94].
**U2AF1**	MDS	7–17	Increased risk of secondary AML and shorter OS, no effect on OS	[95,96].
	Primary myelofibrosis	16	Shorter OS (Q157mutation), no effect on LFS	[96].
	Lung adenocarcinoma	3	Shorter PFS	[97].
**U1**	Hepatocellular carcinoma	5.8	No effect on OS	[98].
	Sonic hedgehog medulloblastoma	8.8	Increased risk of relapse but no effect on OS	[99].
**SRSF2**	MDS without RS	10	Shorter PFS	[85].
	CMML	25–47	No effect on OS	[100].
	Primary myelofibrosis	8.5–17	Shorter LFS and OS	[101].
	Secondary (myeloproliferative neoplasm)AML	16–18	Shorter OS	[102].

Thus, the management of tumor malignancies developed from reoccurrence of the tumor, tumor metastasis, and potential treatment resistance through consideration of PHF5A as a potential therapeutic target for the resolution of clinical crises becomes extremely imperative.

## 6 Participation of PHF5A and its associated complexes as potential therapeutic targets

To retain normal biological cell functioning, regulation of correct spliceosomal assembly is enormously imperative that in turn depends upon the correct alignment of the SF3B1 splicing factor. Frequent mutations of the SF3B1 complex may result in the initiation/formation of various hematological malignancies including chronic myeloid leukemia, chronic lymphocytic leukemia, and myelodysplastic syndrome occurring from the inappropriate branch point selection and dysregulated or abnormal splicing [103,104]. SF3B spliceosomal complex is responsible for the unwinding or recognition of branch point adenosine and selection of respective 3′ splice site [104]. In comparison to DNA sequence regulation, alternative splicing (a phenomenon of generating discrete mRNA and protein isoforms from a particular gene) indicated high streaming and better interventions with its improved potential for the management of cancer. Similarly, for accomplishing individualized cancer treatment; the utilization of splicing modulators for targeting cancer-associated alternative splicing processes may offer innovative and promising therapeutic outputs with the simultaneous designing of cancer vaccines and medicines [82].

Detection of alternative splicing and identification of numerous splicing modulators also explored certain small molecule inhibitors specifically targeting SF3B or SF3B1 complexes. Pladinolide B and Sliceostatin A are reported to exhibit anti-cancer attributes by precisely modulating the PHF5A and SF3B capability of identifying the branch point sequences constituting pre-mRNA. The binding of Spliceostain A or Pladinolide B to the SF3B complex prevents the splicing phenomenon that leads to the inhibition of associated gene expression responsible for cancer prognosis. Inhibition of SF3B in the initial stages induces inhibition of pre-mRNA splicing and consequential leakage/translation of unspliced mRNA [105,106]. Deterrence of SF3B subunit binding to a pre-mRNA sequence enabled Spliceostatin A to prevent the growth, proliferation, and pathogenesis of tumor cells [107].

Antitumor or anti-proliferative capability of Spliceostatin A; a potent SF3B complex inhibitor and a stabilized fermentation-derived product isolated from *Pseudomonas*) has been determined both in cancer cell lines and animal models. Further, enhanced apoptosis and relapse prohibition of therapy-resistant prostate cancer cells resulting from interference of Spliceostatin derivatives with the splicing process has also been ascribed [108,109]. Similarly, the anti-proliferative potential of Spliceostatin A corresponding to the inhibition of the splicing process or spliceosome complex (SF3B1) has been observed in breast cancer (mutant cell lines), cervical cancers and chronic lymphocytic leukemia [107,110,111].

Spliceostatin A is also identified as a promising antineoplastic agent provoking potential anti-angiogenic activity *in-vivo* via its interaction with vascular endothelial growth factor (VEGF). Angiogenesis/neoangiogenesis (a process of formation of newer blood vessels from the hitherto existing microvasculature) is a phenomenon most commonly observed in cancer to accomplish the nutrition and oxygen need of rapidly growing cells. VEGF is a key factor regulating tumorigenesis via the regulation of alternative splicing/VEGF-associated mRNA processing. Spliceostatin A interaction with VEGF along with respective hindrance of VEGF m-RNA splicing or transcription ultimately affects tumor pathogenesis i.e. tumor-associated neo-angiogenesis [112].

Pladienolide B, a potential inhibitor of alternative/m-RNA splicing is known to display excellent antineoplastic activity in numerous xenograft models via splicing impairment through specific targeting of SF3B splicing factor or its SF3B1 subunit. Pladienolide B is a naturally occurring 12-membered macrolide obtained initially from *Streptococcus Platensis* (Mer-11107) during cellular screening performed to evaluate the silencing of hypoxia-induced expression of the VEGF promoter [113,114]. Both *in-vitro* and *in-vivo* studies performed using cancer cell lines and different xenograft models verified the potential anti-proliferative activity of Pladienolide B. Even a highly sensitive xenograft assay (BSY-1) indicated complete eradication of tumor progression upon Pladienolide B administration [114]. Therapeutic attributes of Pladienolide B have been noticed in numerous malignancies including disseminated malignant peritoneal mesothelioma, cervical carcinomas, hematological, colorectal, and pancreatic malignancies [[115], [116], [117]].

Pladienolide B indicated dose-dependent cancer cell apoptosis along with cell cycle arrest in both erythroleukemia cell lines and human cervical carcinoma cells. These findings were associated with the potential binding of a therapeutic agent to the SF3B complex preventing its binding to a branched sequence with a sequential generation of abnormal alternative splicing and ultimate anti-tumor aspects [115,116]. Similarly, Pladienolide B is also reported to prohibit the oncogenic characteristics (tumor cell growth, invasion/migration, and metastasis) of both cancer stem cells and pancreatic adenocarcinoma cells [117]. Correspondingly, Pladienolide B was reported to present anti-proliferative properties associated with the down-regulation of the splicing process in gastric cell lines and patient-derived originally cultured tumor cells with the ultimate progression of in-vivo tumor cells apoptosis and prevention of cellular growth in xenograft tumors [118].

PHF5A; another essential element of the SF3B complex is noticed to be regulated by splicing modulators specifically targeting SF3B or its SF3B1subunit suggesting the existence of a common interaction site. Splicing modulators regulate PHF5A functioning via binding to a common binding site with sequential alteration of the specific splicing pathway. Pladienolide B has been ascribed to exhibit inhibitory effects on PHF5A though it was initially recognized as a potential SF3B inhibitor [29]. Correspondingly, potential inhibition of lung adenocarcinoma cells proliferation and pathogenesis via Pladienolide B indicated potential concentration-dependent anti-proliferative attributes with consequences quite similar to that of PHF5A silencing or knockdown [49].

Singh et al. (2020) described that impairment of RNA splicing attributed to the interference of PHF5A or SF3B splicing components causes DNA strand breaks [119]. Furthermore, PHF5A or SF3B1 depletion provoked improved sensitivity of lymphocytic leukemia cells towards Mitomycin C (DNA crosslinking agent) along with reduced expression of RAD51 (an essential component responsible for DNA double helical strand breaks repair) coupled to elevated levels of y-H2AX. Likewise, analysis of the in-vitro pharmacological activity of Pladienolide B also indicated similar outcomes suggesting a strong correlation of DNA damaging ingredients (Pladienolide B) with PHF5A or SF3B complex [119].

Similarly, in Glucocorticoid resistant lymphoblastic leukemia, the therapeutic effectiveness and potential attention of Pladienolide B as a promising splicing modulator were prompted due to the enhanced sensitivity of lymphoblastic leukemia cell line (CEM/R30 dm) to Pladienolide B [120].

Often PHF5A/SF3B mutation causes DNA damage primarily observed in patients suffering from myeloid malignancies particularly myelodysplastic syndrome [119,121]. Further, a mutation in the PHF5A/SF3B1 sub-unit of the SF3B complex was established through genome sequencing and resistant strains screening of Spliceostatin A and Pladienolide B splicing modulators. Screening analysis negotiated several mutants responsible for the development of resistance against splicing modulators including PHF5A-Y36C, SF3B1–K1071, SF3B1-R1074, and SF3b1-V1078. Yet, resistance emerged from PHF5A-Y36C mutant demonstrated absolute inhibition of Spliceostatin A and Pladienolide B functioning via alteration of intron retention/exon skipping profile provoked by these splicing modulators [29].

Resistance occurrence against Pladienolide B is occasionally associated with variation in the conformation of SF3B complex from open form to a closed state with eventual variation in normal alternative splicing [122]. In contrast, enhanced sensitivity of PHF5A-R38C mutant toward Pladienolide analogs has also been displayed [123]. Thus, in comparison to PHF5A, PHF5A mutants provoke biologically distinctive consequences toward splicing modulators.

## 7 Conclusion

In spite of great advancement in cytotoxic chemotherapeutics, therapeutic management of tumor malignancies remained unsuccessful due to certain intervening aspects such as cytotoxicity of chemotherapeutic agents used towards normal healthy cells leading to poor survival rate and patient death in case of metastatic tumor. Secondly, drug resistance offered by tumor cells against cytotoxic agents most resulting from the oncogenic mutations and stimulation of several signaling pathways regulating the up work of different compensatory mechanisms enabling the carcinogenic cells to ultimately accomplish their nutritional and oxygen demand. Additionally cancer relapse associated with carcinogenic cells metastasis to the other body tissue and poor drug permeability and biocompatibility factors present challenging issues in the cancer treatment. Considering the associated factors site specific tumor therapy is preferred via selective targeting of interconnected network responsible for cancer pathogenesis. Overexpression of different proteins or biomarkers helped researchers to utilize them as potential therapeutic drug targets for tumor therapy. Targeting therapeutic biomarkers for the cancer therapy might be successful in improving overall treatment outcomes and patient survival rate via presenting minimum damage to healthy cells.

PHF5A consideration as a promising therapeutic biomarker for the management of cancer malignancies is imperatively attributed to its pivotal role in cancer initiation and progression along with its effective participation in spliceosome and non-spliceosome related attributes responsible for the regulation of CSCs. PHF5A targeting via particular site-specific splicing modulators might be helpful in the recognition of unique splicing modulators together with the acquisition of innovative structure-based drug design for naturally available therapeutic moieties. PHF5A assessment as a potential biomarker and splicing component attains advanced consideration for a) determination of its structural, biological, and functional attributes b) analysis of mutation sites, and c) determination of underlying molecular mechanism responsible for tumor propagation and progression for designing of site-specific therapeutic moieties. The current review is aimed to explore novel techniques or ideas that might be helpful to practically bring advancement in cancer therapy at the clinical level.

## Author contribution statement

All authors listed have significantly contributed to the development and the writing of this article.

## Data availability statement

Data included in article/supplementary material/referenced in article.

## Declaration of competing interest

The authors declare that they have no known competing financial interests or personal relationships that could have appeared to influence the work reported in this paper
